# ADAR Mediated RNA Editing Modulates MicroRNA Targeting in Human Breast Cancer

**DOI:** 10.3390/pr6050042

**Published:** 2018-04-25

**Authors:** Justin T. Roberts, Dillon G. Patterson, Valeria M. King, Shivam V. Amin, Caroline J. Polska, Dominika Houserova, Aline Crucello, Emmaline C. Barnhill, Molly M. Miller, Timothy D. Sherman, Glen M. Borchert

**Affiliations:** 1 Department of Biology, University of South Alabama, Mobile, AL 36688-0002, USA;; 2 Department of Pharmacology, USA College of Medicine, Mobile, AL 36688-0002, USA; dh1001@jagmail.southalabama.edu

**Keywords:** ADAR, breast, cancer, inosine, microRNA, microRNA targeting, RNA editing

## Abstract

RNA editing by RNA specific adenosine deaminase acting on RNA (ADAR) is increasingly being found to alter microRNA (miRNA) regulation. Editing of miRNA transcripts can affect their processing, as well as which messenger RNAs (mRNAs) they target. Further, editing of target mRNAs can also affect their complementarity to miRNAs. Notably, ADAR editing is often increased in malignancy with the effect of these RNA changes being largely unclear. In addition, numerous reports have now identified an array of miRNAs that directly contribute to various malignancies although the majority of their targets remain largely undefined. Here we propose that modulating the targets of miRNAs via mRNA editing is a frequent occurrence in cancer and an underappreciated participant in pathology. In order to more accurately characterize the relationship between these two regulatory processes, this study examined RNA editing events within mRNA sequences of two breast cancer cell lines (MCF-7 and MDA-MB-231) and determined whether or not these edits could modulate miRNA associations. Computational analyses of RNA-Seq data from these two cell lines identified over 50,000 recurrent editing sites within human mRNAs, and many of these were located in 3’ untranslated regions (UTRs). When these locations were screened against the list of currently-annotated miRNAs we discovered that editing caused a subset (~9%) to have significant alterations to mRNA complementarity. One miRNA in particular, miR-140–3p, is known to be misexpressed in many breast cancers, and we found that mRNA editing allowed this miRNA to directly target the apoptosis inducing gene *DFFA* in MCF-7, but not in MDA-MB-231 cells. As these two cell lines are known to have distinct characteristics in terms of morphology, invasiveness and physiological responses, we hypothesized that the differential RNA editing of *DFFA* in these two cell lines could contribute to their phenotypic differences. Indeed, we confirmed through western blotting that inhibiting miR-140–3p increases expression of the *DFFA* protein product in MCF-7, but not MDA-MB-231, and further that inhibition of miR-140–3p also increases cellular growth in MCF-7, but not MDA-MB-231. Broadly, these results suggest that the creation of miRNA targets may be an underappreciated function of ADAR and may help further elucidate the role of RNA editing in tumor pathogenicity.

## 1. Introduction

**Figure 1.**
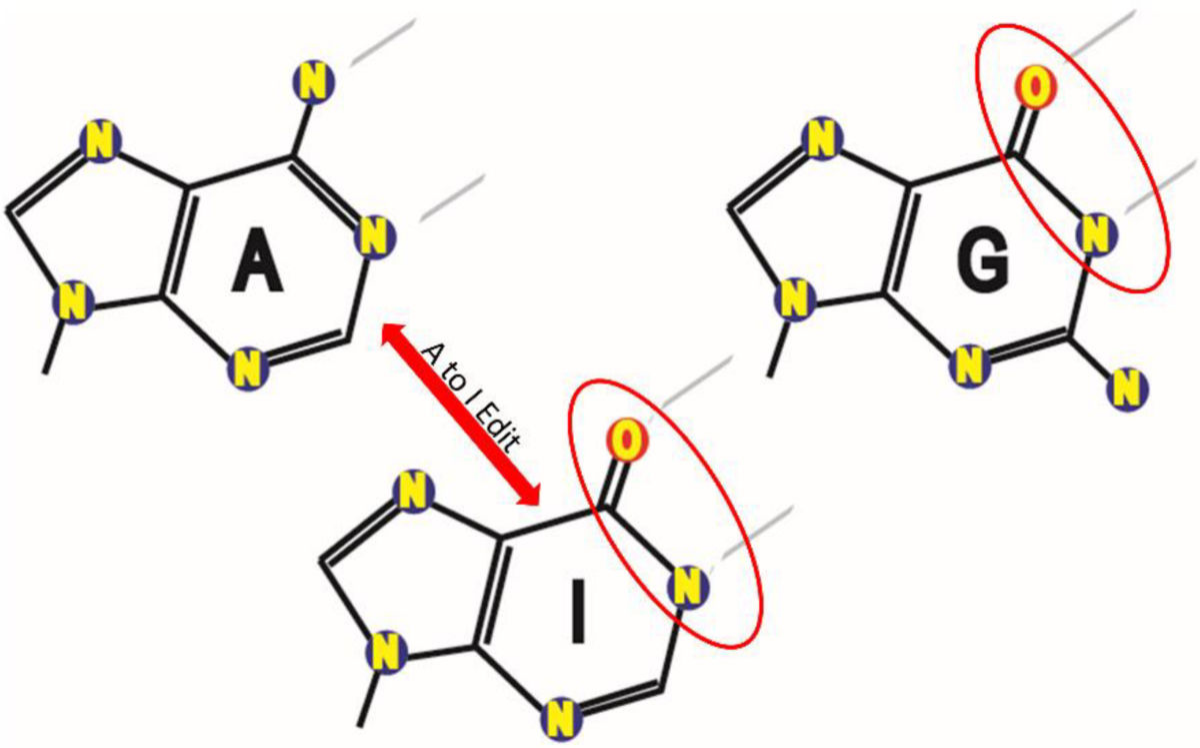
ADARs deaminate adenosine to inosine, potentially altering miRNA complementarities. A cartoon depicting adenosine (**left**), deaminated adenosine (inosine, in **center**), and guanine (**right**).

Transcript variation at the single nucleotide level is increasingly being found to have widespread occurrences within the transcriptome with fundamental roles in numerous biological processes including development and disease. Specifically, several independent studies have reported that there are hundreds of thousands of RNA editing sites catalyzed by the enzyme ADAR (adenosine deaminase acting on RNA) within human mRNAs [1–6]. Editing via ADAR is characterized by the conversion of the nucleic acid adenosine to inosine via deamination at the C6 position [7] (Figure 1). Since inosines have been shown to preferentially bind to cytosines, functionally the ADAR-catalyzed editing changes an ‘A’ to a ‘G’ in the transcript sequence [7]. Interestingly, the vast majority (>99%) of editing sites occur in the UTRs of primate-specific Alu elements [8–10], likely due to the common occurrence of two oppositely oriented Alus located in the same pre-mRNA pairing together to produce the long and stable double-stranded RNA structure that is required for ADAR to bind. As the ability to convert nucleotides adds a great deal of functionality to the transcriptome, it is not surprising that it has fundamental roles in many cellular activities. Editing events within mRNA coding for various neuroreceptors, such as serotonin and glutamate, have been intensively detailed in an array of organisms from flatworms to primates and found to be critical for routine neural activity [11]. With regards to gene regulation, sequence editing of RNA has widespread implications, including splice site alteration, localization and nuclear retention, and modification to the RNA secondary structure itself [12]. Further, given these abundant roles for ADAR editing in routine cellular function, it should also not be surprising that dysfunction of this important mechanism can have detrimental effects and, indeed, an increasing number of reports indicate a strong correlation between altered ADAR activity and a variety of pathologies. Specifically, because ADAR has been shown to be such an integral player in apoptotic regulation and cellular differentiation, cancer is of especially heightened interest and, in fact, more and more evidence is pointing to dysregulation of the editing process being a major factor in tumorigenesis [13–18].

Editing events can also have widespread effects on the gene regulatory ability of noncoding RNAs, such as miRNAs [17,19]. MiRNAs are small regulatory RNA molecules roughly 20 to 23 nucleotides in length that regulate cell processes by binding to their target mRNAs and inhibiting translation [20]. MiRNAs are initially transcribed as primary miRNAs (pri-miRNAs) consisting of several thousand nucleotides in length that are then processed by into mature miRNAs by the enzymes Dicer and Drosha before entering the RNAi gene silencing complex where they regulate gene expression by binding to the 3’ UTR of their mRNA targets via complimentary base pairing and silencing the gene by either repressing translation of the mRNA or triggering its degradation [21]. MiRNAs have been found to play a role in numerous cellular processes, from cell cycle control and apoptosis regulation to hormone production and immune response [22]. Importantly, misexpression of miRNAs has been implicated in a number of different disease states ranging from cardiovascular [23] and neurological disorders [24] to many types of cancer [25]. Having been associated with such a wide array of processes and pathologies, these molecules have garnered increased attention recently as investigators begin to evaluate their potential utility as biomarkers and therapies.

While these two mechanisms are fairly well understood independently, only recently have reports profiled the functional connection between RNA editing and miRNAs. For example, it has been shown that ADAR1 forms a complex with Dicer through direct protein interactions and enhances global miRNA processing [26]. Further, ADAR deamination of pri-miRNA transcripts can cause alterations to their structural conformations and subsequent maturation and processing by Drosha and Dicer [27,28]. In addition, while any editing of miRNA transcripts can have functional implications, arguably the most critical changes are to the seed regions of the miRNAs as this can drastically alter the set of genes able to be regulated [29]. This is especially true in cancer where altered miRNA regulation of oncogenes and tumor suppressors can lead to tumor formation [17]. Importantly, it should be noted that, in addition to editing the miRNA transcript, ADAR can also edit the 3’ UTR of target mRNAs. This modification dramatically increases the interplay between miRNAs and their targets by allowing a different set of miRNAs to regulate a given mRNA depending on if the transcript has been edited or not. Unfortunately, while the effects of editing the miRNA transcripts themselves have been well documented, this opposite effect of editing mRNAs in regions complementary to miRNA seeds is less understood. However, a number of reports suggesting this plays a significantly underappreciated role in miRNA targeting have surfaced within the last year [30–33]. To examine this, our study identified edit sites within two breast cancer cell lines (MCF-7 and MDA-MB-231) and analyzed the effect these edits had on subsequent regulation by miRNAs.

## 2. Materials and Methods

### 2.1. NGS Sequencing of MCF-7 and MDA-MB-231

Two breast cancer cell lines (MCF-7 and MDA-MB-231) grown under standard procedures were obtained from colleagues at the Mitchell Cancer Institute (Mobile, AL, USA). RNA was isolated and suspended in Trizol per standard manufacture protocol before being shipped to Otogenetics (Otogenetics Corporation, Atlanta, GA, USA) for commercial next-generation sequencing on an Illumina HiSeq2000 sequencer. Two RNA-Seq protocols were requested: (1) a total polyA selected RNA-Seq to provide mRNA transcripts, and (2) a small size selected RNA-Seq to provide small RNAs ranging from 17 to 35 nt in length. Raw paired-end reads were received totaling around 6 billion base pairs per cell line. Reads were uploaded to the NCBI Sequence Read Archive (SRA) and assigned the project number SRP101635.

### 2.2. Identification of A-to-G Edits in Breast Cancer Cells

Reads from the polyA selected RNA-Seq were filtered for low-quality reads and adapter contamination using Trimmomatic [34] and then aligned to the GRCh38 human reference genome using TopHat [35] (one mismatch allowed per alignment, only unique mappings reported). Edit sites were identified using the ‘mpileup’ command of SAMtools [36] which generates a VCF file containing location information for observed variations between the reads and the reference. All identified variations other than A-to-G and T-to-C were removed, and the remaining locations were cross-referenced with the dbSNP database to exclude variations that are known SNPs. To be considered a probable edit, at least 10% of transcriptome reads were required to differ from the reference genome at the edit position (with a minimum of 30 total reads).

### 2.3. Computational Identification of MiRNAs Biased towards Editing

**Figure 2.**
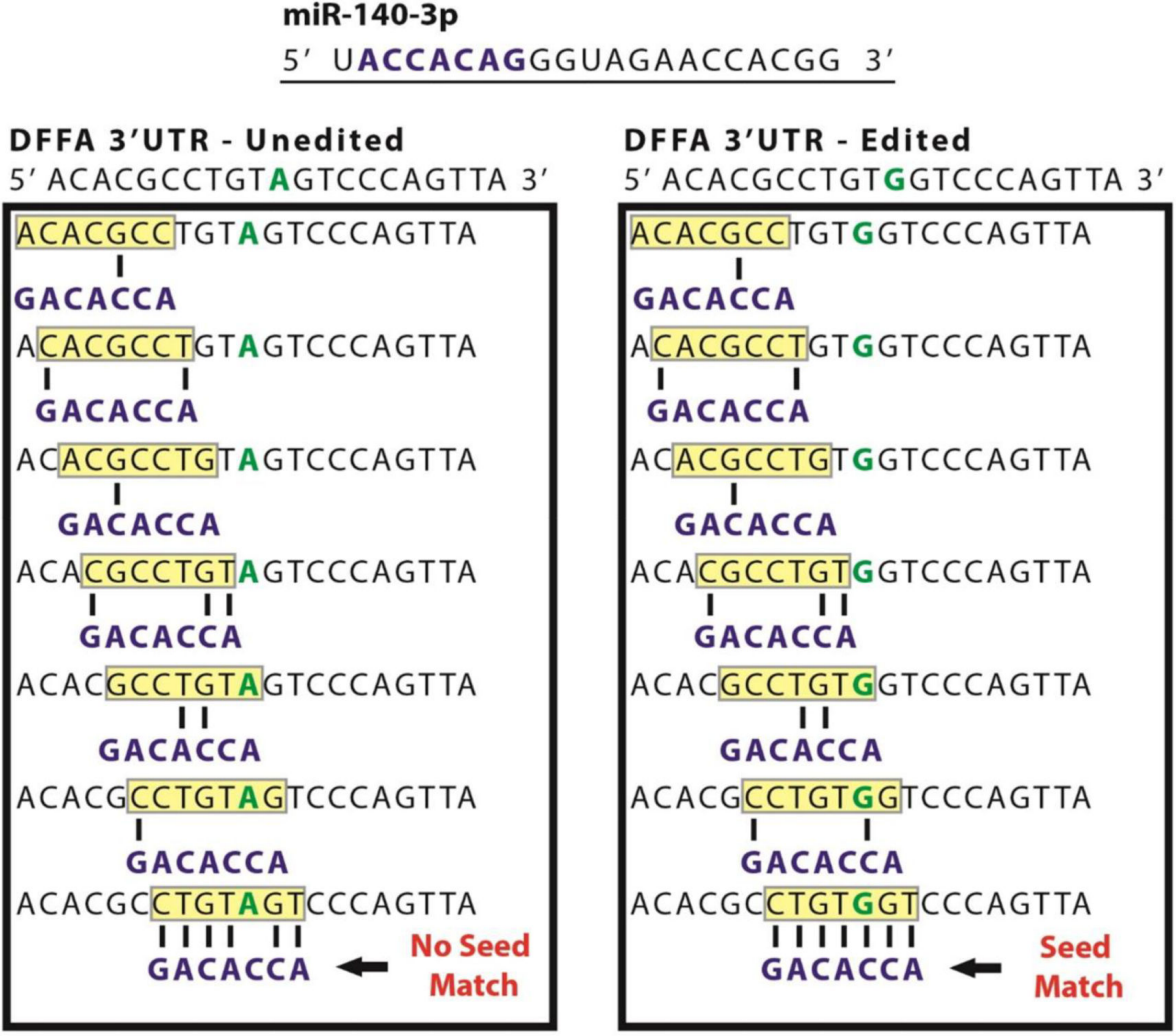
Effect of RNA editing on DFFA. A representative deamination site (green) occurring in the 3’ UTR of DNA fragmentation factor α (DFFA) is shown in both the unedited (**left**) and edited (**right**) state. The seed of miR-140–3p (blue) was screened using a sliding windows approach (depicted with a yellow box) against all possible seed matches within the DFFA sequence. Complimentary base pairing is indicated by the black lines.

The list of remaining putative edit sites (plus the sites identified in a previous study [37]) were used to generate a dataset consisting of two files each containing 201 bp sequences (edit site plus/minus 100 bp flanking sequences from the human reference genome). One file contained an ‘unedited’ version of the transcript with the edit site corresponding to the reference genome, and the other file contained an ‘edited’ version where the central site was edited. An in-house program written in Java was used to compare the reverse complement of the 7 nt seed sequences from all 2588 known human miRNAs in miRbase [38] to each possible 7-mer sequence within the generated dataset using a sliding window approach that counted perfect seed matches and recorded the position of each match in an Excel file (illustrated in Figure 2). Both the edited and unedited set of transcripts were analyzed for comparison, and after statistical analysis those miRNAs whose total number of seed matches increased or decreased significantly (10-fold or higher) in one set or the other were said to be biased towards editing.

### 2.4. Small RNA-Seq Analysis

To generate miRNA expressions data, reads from the small RNA-Seq experiment were aligned to known miRNA transcripts using the BLAST+ [39] sequence aligner. In order to be reported as valid the alignment was required to be over 99% similar with no more than one mismatch over 36 base pairs.

### 2.5. Cell Growth Assay

MDA-MB-231 cells were first transfected with either 100 nmol/L of miR-140 antagomir (Anti-140) (Cat # C-301055–01-0005, GE Healthcare Dharmacon, Chicago, IL, USA) or scrambled negative control (Ctrl-140) (catalog number CN-001000–01-05, Dharmacon) using Lipofectamine (Life Technologies, Carlsbad, CA, USA) according to the manufacturers protocol. Cell number was determined by trypan blue staining and manual counting at 24, 36, and 48 h post-transfection. Growth was determined as the relative cell number compared with vehicle-treated (0.1% DMSO) controls.

### 2.6. Western Blot Analysis

Following transfection of cells with anti-miRNAs, at 36 h existing media was replaced with lysis buffer containing protease inhibitors, incubated for 15 min at 4 °C, and then transferred to tubes. The cell proteins were electrophoresed through an 8% SDS–polyacrylamide gel and transferred to polyvinylidene fluoride membranes for the immobilization of the proteins. The membranes were blocked for 1 h in 2% non-fat milk in phosphate-buffered saline containing 0.05% Tween-20 surfactant and then washed and incubated with primary DFFA (ICAD) antibody (LF-PA0058, Thermo Scientific, Rockford, IL, USA) overnight at 4 °C. Following subsequent washing and incubation with goat anti-rabbit peroxidase-conjugated secondary antibody the immunoreactive bands were visualized and quantified using a Flurochem densitometer for the reporting of the protein levels.

## 3. Results

In order to characterize transcriptional differences between MCF-7 and MDA-MB-231 cells, RNA was isolated from each and split into “mRNA” (>200 nt RNAs) and “small RNA” (<200 nt RNAs including mature miRNAs) fractions. These samples were commercially sequenced resulting in over 2 billion nucleotides of small RNA reads and roughly 6 billion nucleotides of the longer mRNA reads.

### 3.1. Identification of RNA Edit Sites

**Figure 3.**
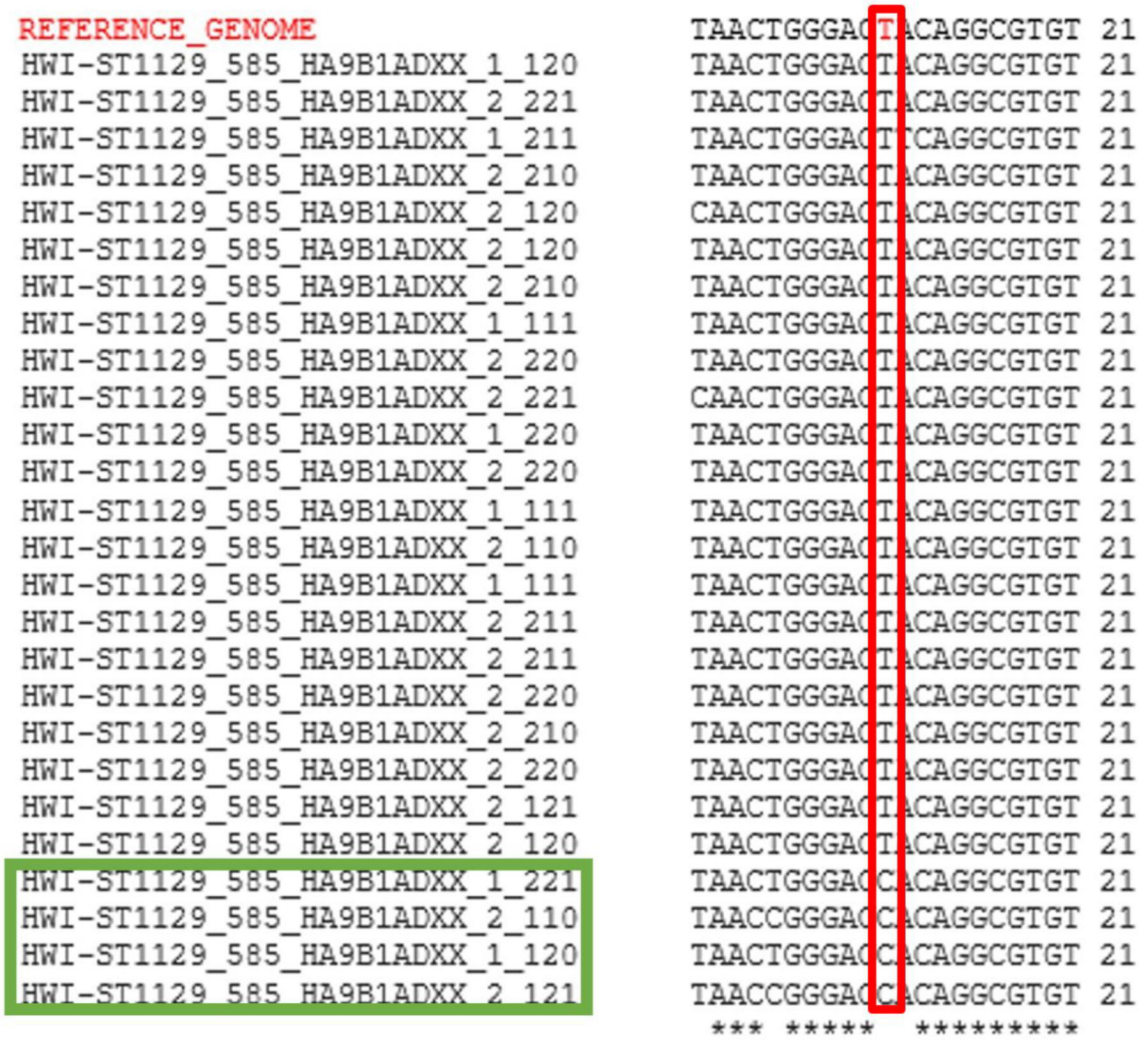
Alignment of RNA-Seq reads to the human genome. Poly(A) selected RNA from two breast cancer cell lines (MCF-7 and MDA-MB-231) were sequenced with an Illumina Hi-Seq to provide high coverage mRNA transcripts. These transcripts were then compared to reference genome (top in red), with mismatches indicating a possible site of editing activity. Here one such site is shown within the red box, with mismatched reads outlined in green. Alignment was generated using ClustalW (http://www.genome.jp/tools-bin/clustalw) [40].

Identification of putative RNA edit sites within each of the two cell lines was performed by mapping RNA-Seq reads to the GRCh38 human reference genome. As read alignments are reported with respect to the leading strand of the reference genome, a putative edit site would appear as an A-to-G mutation if the read arose from the forward strand, or a T-to-C mutation in the case of the reverse strand (Figure 3). In all, 19,462 unique edit sites were identified in MCF-7 and 35,090 sites were found in MDA-MB-231 (Table S1). That said, we found reads containing edits differed from the reference genome at the edit position 51.8% of the time in MCF-7s on average and 49.8% of the time in MDA-MB-231s.

### 3.2. MiRNAs Biased towards Editing

Subsequent identification of miRNAs whose set of predicted target mRNAs were significantly affected due to our identified mRNA deaminations was achieved by screening the ‘seed’ regions from all 2588 currently-annotated human miRNAs in miRBase [38] against our full set of putative edit sites and an independently-generated publicly-available set of >12,000 A-to-I human edit sites [37] (Figure 2). Cataloging all of a miRNA’s seed matches in both edited and unedited transcripts identified a subset whose mRNA target sets were significantly altered due to RNA editing (Tables 1, S2 and S3). In total, 206 miRNAs were shown to have altered target sites caused by ADAR-mediated single nucleotide mutations. Interestingly, we found that 86 of these miRNAs appeared to specifically target edited sequences and participate in regulations nonexistent prior to editing (Table S2) and, conversely, that the targets sites of the other 120 miRNAs were instead ablated upon ADAR editing due to a loss of sequence complementarity to their predicted mRNA targets (Table S3). As such, in order to ascertain whether any of these miRNAs were being actively expressed in our two cell lines, we next performed an expression analysis using our small RNA-Seq reads via BLAST+ [39]. Reads were aligned to known miRNAs, limited to only the highest scoring alignment per read, and required to be 100% identical to annotated miRNAs. Using these criteria, we identified 20 miRNAs for further evaluation based on their relative high expressions (>50 reads per million) in both MCF-7 and MDA-MB-231 (Table 1).

### 3.3. MiR-140 Is Able to Target DFFA in MCF-7 but not MDA-MB-231

**Figure 4.**
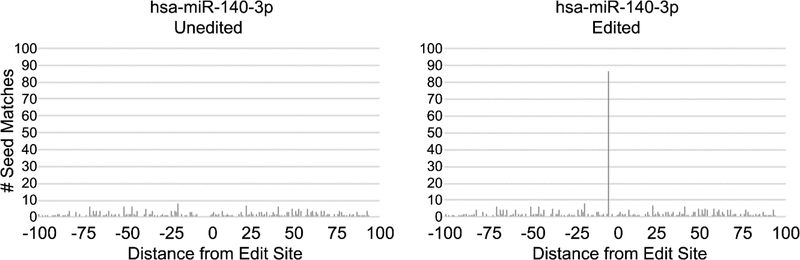
A-to-I edits create novel target sites for miR-140–3p. mRNA sequences from the edit sites previously identified [37] (each consisting of a central A-to-I deamination and 100 nt flanks) were screened for complementarity to human miRNAs. The graphs represent all miR-140–3p seed matches occurring at each possible position within both the unedited (**left**) and edited (**right**) states.

**Figure 5.**
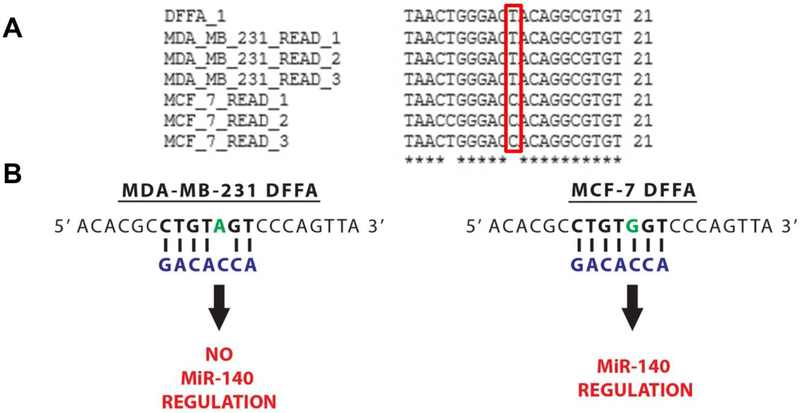
MiR-140 can regulate *DFFA* in MCF-7, but not MDA-MB-231. (**A**) Alignment of 21 nt segments of six RNA-Seq reads (three from each cell line) to a portion of the apoptosis inducing gene DFFA. Our edit identification algorithm identified an A-to-G edit site at basepair 10,460,668 on Chromosome 1, and corresponding reads mapping to that location were extracted and trimmed to 21 bp (edit site plus/minus 10 bp flanking regions). Edit location is outlined in red. The alignment was generated via ClustalW [40]. (**B**) Illustration showing complimentary base pairing between the miR-140 seed (blue) and the DFFA gene in both cell lines. The edit site is indicated in green.

Next, after a thorough examination of the subset of miRNAs whose set of predicted target mRNAs were significantly affected by deamination in our cell lines, we selected miR-140–3p for a detailed experimental examination. Importantly, we found miR-140–3p was highly expressed in both cell lines and, notably, its set of target mRNAs was found to be significantly altered by RNA editing in MCF-7 cells, but not in the MDA_MB-231 cells. Importantly, we found A-to-G mutations caused dramatic changes to miR-140’s set of predicted mRNA target sites in MCF-7s, with deamination events leading to the creation of 91 new putative target sites in 34 mRNAs. Of note, through utilizing strategies we previously employed to successfully identify sites created in a publicly-available set of >12,000 A-to-I human edit sites [37,41] (Figure 4), we identified a particularly interesting target site created for miR-140–3p in MCF-7 cells—DNA fragmentation factor alpha (DFFA), also known as inhibitor of caspase-activated DNase (ICAD) (Figure 5). As the principle function of DFFA is to trigger DNA fragmentation during apoptosis, we hypothesized that the miRNA-mediated downregulation of this gene specifically in MCF-7 cells might directly contribute to their characteristically lower rate of cellular proliferation as compared to MDA-MB-231s.

### 3.4. Inhibiting miR-140–3p Increases DFFA Expression in MCF-7

**Figure 6.**
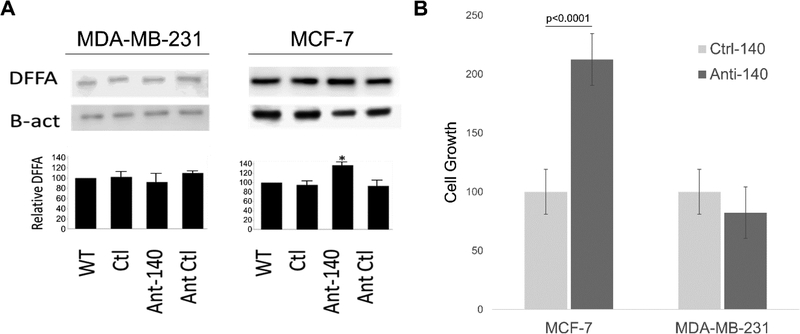
Depletion of DFFA protein expression and the effect of miR-140–3p on cellular growth. (**A**) Representative blots for DFFA and β-actin (loading control) are shown (n = 3). The miRNA is able to bind and regulate the *DFFA* gene in MCF-7, but not in MDA-MB-231 due the presence of an A-to-I edit. WT, wild type; Ctl, empty lipo transfection; Ant-140, miR-140 antagomir; Ant-Ctl, random antagomir. (**B**) Cell growth assay examining effects of transfecting a miR-140 inhibitor in both cell lines. Five microscopic fields randomly chosen from each assay were counted individually, and the statistical significance between treatment and control determined by *t*-test.

In order to determine if miR-140–3p directly regulates the endogenous expression of DFFA, we performed DFFA Western blots (Figure 6A) to examine the effects of introducing a specific miR-140–3p antagomir as compared to a non-specific control. Excitingly, although we found a marked increase of DFFA levels following miR-140–3p inhibition in MCF-7s (where a target site is created by ADAR deamination), we found no appreciable effect of inhibiting miR-140–3p in MDA-MB-231s (in which DFFA does not undergo deamination). Furthermore, qPCR analysis of DFFA expression found no effect on DFFA mRNA levels following miR-140–3p inhibition in either cell line (data not shown) confirming miR-140–3p regulates DFFA post transcriptionally.

### 3.5. Inhibiting miR-140–3p Increases MCF-7 Cellular Proliferation

We next examined the effects of inhibiting miR-140–3p on cellular growth and similarly found cellular growth was largely unaffected by decreased miR-140–3p levels in MDA-MB-231, whereas we found there was over a 110% increase in MCF-7 cellular growth following miR-140–3p depletion at 24 h post transfection (Figure 6B). Importantly, these results strongly agree with our examination of *DFFA* regulations and further support the idea that miR-140–3p mediated downregulation of DFFA specifically in MCF-7 cells directly contributes to the characterized differences of these two cell lines in cellular growth.

## 4. Discussion

ADAR-mediated RNA editing is well characterized as having dramatic effects on a multitude of cellular processes [11,18,42,43]. However, the molecular mechanisms through which ADAR editing confers these effects remain largely undefined. That said, ADAR editing of miRNA transcripts has now been shown to affect their regulatory ability, in some cases leaving them unable to bind to their target transcripts and in others leading to unintended inhibition of new targets altogether [17,19,44]. To add to the relationship between A-to-I editing and miRNAs, we have now successfully shown that mRNA editing can also affect miRNA targeting by changing the complementarity between a 3’ UTR binding site and the seed region of a miRNA. Results from our analysis strongly suggest that A-to-I editing is routinely employed to modify mRNA complementarities to a specific subset of 233 human microRNAs currently annotated in miRBase [38]. Interestingly, for 86 of these miRNAs ADAR editing leads to the generation of new regulatory targets, whereas A-to-I editing conversely results in a significant loss of complementarity to mRNAs and, therefore, a loss of putative targets for the other 120 miRNAs. We find these two subsets of ADAR editing-related miRNAs to be completely distinct—86 specifically targeting edited mRNAs and 120 specifically targeting unedited mRNAs (or whose regulation is blocked by editing). This latter observation is notable as the ability of ADAR to destroy mRNA targets has not been previously reported and is in direct contrast to previous work that suggested ADAR editing could likely only create targets for miRNAs [41].

Based on these results, we believe that the generation of novel miRNA regulatory networks is a critical function of ADAR editing, and, notably, that dysregulated editing may create susceptibilities that allow tumorigenesis and tumor progression to occur. Corroborating this idea, several studies have already established a clear precedent for ADAR activity being implicated in cancer biology. Recently, Chen et al. [15] described direct involvement of ADAR editing in human hepatocellular carcinoma (HCC), showing how the transcripts of an oncoprotein degrader and confirmed contributor to HCC pathology, antizyme inhibitor 1 (*AZIN1*), are modified at specific sites by ADAR1, and that ADAR1 is commonly upregulated in HCC patient tumors resulting in even higher *AZIN1* editing frequency and poorer prognosis. In addition, the authors were able to successfully demonstrate that higher levels of edited *AZIN1* promoted an increased incidence of tumor formation and invasive ability. Over-editing of *AZIN1* has also been implicated in other cancers, such as esophageal squamous cell carcinoma [13]. Other recent studies suggest that ADAR1 might also play a pathogenic role in chronic myeloid leukemia (CML). Jiang et al. [14] have recently shown that overexpression of ADAR1 in cultured blood progenitor cells can promote reprogramming of myeloid progenitor cells resulting in heightened hematopoietic differentiation toward the myeloid lineage. Increased ADAR1 levels were repeatedly found in CML patient samples leading the authors to speculate that ADAR played a causal role. In fact, a related study recently found CML could not be induced in mice following a bone marrow transplant of marrow cells carrying an ADAR deletion suggesting ADAR1 may be essential for leukemia cell survival [14].

In contrast to the previous examples linking hyper-editing to malignancy, the opposite scenario, hypo-editing, has also been implicated as contributing to various cancers, specifically in relation to miRNAs. For instance, it has been shown by Choudhury et al. [17] that reduced editing of miR-376a promotes glioblastoma cell invasion in orthotopic glioma. Normally-edited miR-376a targets and suppresses the receptor for the autocrine motility factor (AMF) that stimulates tumor motility via base pair complementarity with the 3’ UTR of the AMF receptor mRNA; however, when unedited, the miRNA loses this ability. It was also demonstrated that unedited miR-376a binds to the 3’ UTR of the *RAP2A* mRNA transcript (coding for a protein known to suppress glioblastoma cell invasion), causing the RAP2A protein’s function to be inhibited. This report does an excellent job of demonstrating how ADAR-induced single base pair changes in miRNAs can alter their target specificity and ultimately lead to pathologically significant ramifications. Further, while it is clear that RNA editing can be fundamentally linked to cancer via sequence alteration and the expression/repression of oncogenes, there is also evidence of involvement in other tumorigenic pathways. For instance, a correlation has been shown between reduced editing of Alu elements and multiple tumors, including brain, prostate, lung, and kidneys [14,18]. Additionally, chronic inflammation related to viral infection has been previously implicated in tumorigenesis and this may be due, in part, to overexpression of ADAR1 mediated by inflammation [45]. Of note, in this work we identify 19,462 unique edit sites in MCF-7 cells versus 35,090 unique sites in MDA-MB-231s suggesting generally higher ADAR1 activity in this more aggressive breast cancer cell line.

Importantly, the work presented here represents the most comprehensive of only a handful of analyses of the effects of mRNA A-to-I editing on miRNA targeting published to date [30–32], and represents only the second ever experimental evidence indicating that the modulation of miRNA targeting through ADAR editing may directly contribute to breast cancer pathology [33]. When taken together, this report along with recently published studies suggesting mRNA editing can alter microRNA regulations [30–33] (all published within the last few months) strongly suggest that the participation of A-to-I editing in directing microRNA targeting is currently significantly underappreciated.

That said, our analysis of the RNA editing data from two breast cancer cell lines demonstrate that miR-140–3p is able to regulate the apoptosis inducing gene *DFFA* in MCF-7 but not in MDA-MB-231. DFFA is the larger of two protein subunits that comprise caspase-activated DNase (CAD) and, when bound to CAD, DFFA inhibits its ability to degrade DNA and condense chromatin, but during apoptosis caspase-3 cleaves DFFA resulting in DNA fragmentation [46]. As misexpression of an apoptotic contributor can have significant ramifications in terms of tumor development, the differential regulation of DFFA by miR-140 between our two cell lines is highly intriguing, especially as numerous reports have previously implicated a role for miR-140 in breast malignancy [47–49]. That said, the two cell lines involved in this study, MCF-7 and MDA-MB-231, have very distinct characteristics in terms of morphology, invasiveness, and physiological responses. While they are both adenocarcinomas (cancers of the breast epithelium tissue that originated in the mammary gland), the MCF-7 line was derived from an in situ carcinoma where the cancerous cells had not yet invaded surrounding tissues. These cells are weakly invasive, luminal epithelial-like, and are hormone responsive, requiring noticeably less aggressive therapies [50]. In contrast, the highly-invasive, fibroblast-like MDA-MB-231 line was derived from a metastatic carcinoma and is a triple-negative breast cancer making it highly chemoresistant and, thus, significantly more difficult to treat [51]. When taken in conjunction with reports of elevated ADAR activity in many breast cancers, it is feasible to assume that RNA editing could contribute to some of the characteristic phenotypic differences observed between these two cell lines. Excitingly, we suggest the work presented here strongly supports this as we find ADAR editing directly mediates the regulation of *DFFA* in MCF-7s whereas the absence of *DFFA* editing in MDA-MB-231 conversely disallows *DFFA* regulation by miR-140–3p in these cells. Simply put, we find miR-140 is able to bind and regulate *DFFA* due to editing in MCF-7s, so inhibition of the miRNA increases growth. As it is unable to bind in MDA-MB-231, no effect is seen. As such, it is tempting to speculate that the differential regulation of *DFFA* by miR-140–3p between these two breast cancer lines directly contributes to their observed differences in cellular proliferation and cellular survival (Figure 6). That said, miR-140–3p undoubtedly regulates multiple mRNAs and the observed effects on cellular growth may be mediated through more than DFFA restriction alone. Of note, Salem et al. [52] recently demonstrated that transfecting several breast cancer cell lines with miR-140–3p isoform mimics commonly resulted in a decrease in breast cancer cell viability (nicely complementing the increased cellular growth we observe in MCF-7s following transfection of miR-140–3p inhibitor). Additionally, and also in agreement with our findings, this group similarly observed no change in MDA-MB-231 viability following manipulation of miR-140–3p levels via transfection of a miR-140–3p mimic.

While this work represents the first direct indication of a contributory role for A-to-I editing in modulating miRNA targeting in malignancy, we suggest the repeated observation of a correlation between altered ADAR activity and various pathologies suggests altered miRNA regulations due to alterations in A-to-I profiles may represent a significant currently underappreciated contributor to an array of pathologies. Perhaps of broader importance. However, our findings lead us to believe that many miRNA targets can only be identified by analyzing expressed sequences, and that accurate miRNA target prediction may ultimately require analyzing transcriptomes and not genomes.

## Supplementary Material

Supplemental Table 1

Supplemental Table 2

Supplemental Table 3

## Figures and Tables

**Table 1.** List of top 10 miRs where ADAR editing of mRNAs alters complementarity to miR seed regions and either (**A**) creates novel target sites for regulation or (**B**) destroys predicted target sites. In addition to altered edit complementarity, microRNAs included were also required to be present at >50 reads per million in MCF-7 and MDA-MB-231 small RNA-Seq datasets.


miR	miRBase ID	Seed (RC)	Targets (Edited)	Targets (Unedited)	Expected

**A**					

hsa-miR-513a-5p	MIMAT0002877	CCTGTGA	258	0	0.63
hsa-miR-450b-3p	MIMAT0004910	GATCCCA	252	4	0.79
hsa-miR-769–3p	MIMAT0003887	GATCCCA	252	4	0.79
hsa-miR-6089	MIMAT0023714	CGGCCTC	219	0	3.83
hsa-miR-4691–3p	MIMAT0019782	GTGGCTG	181	0	1.16
hsa-miR-3189–3p	MIMAT0015071	CCCAAGG	140	5	0.48
hsa-miR-140–3p	MIMAT0004597	CTGTGGT	139	0	1.11
hsa-miR-3065–3p	MIMAT0015378	GGTGCTG	118	0	0.5
hsa-miR-3940–3p	MIMAT0018356	CCGGGCT	111	0	0.72
hsa-miR-3680–3p	MIMAT0018107	ATGCAAA	108	2	0.82

**B**					

hsa-miR-5089–5p	MIMAT0021081	AATCCCA	0	644	21.39
hsa-miR-6504–3p	MIMAT0025465	CTGTAAT	58	587	19.93
hsa-miR-6506–5p	MIMAT0025468	ATCCCAG	18	377	21.57
hsa-miR-619–5p	MIMAT0026622	ATCCCAG	18	377	21.57
hsa-miR-4775	MIMAT0019931	AAAATTA	0	351	19.37
hsa-miR-4735–5p	MIMAT0019860	AAATTAG	6	305	17.31
hsa-miR-6514–3p	MIMAT0025485	ACAGGCA	10	216	9.59
hsa-miR-4794	MIMAT0019967	TAGCCAG	10	173	8.05
hsa-miR-664a-5p	MIMAT0005948	TAGCCAG	10	173	8.05
hsa-miR-1273e	MIMAT0018079	TCAAGCA	2	169	5.22

