# Supplementary material for: ADAR Mediated RNA Editing Modulates MicroRNA Targeting in Human Breast Cancer
Source: Processes (Basel). Author manuscript; Available in PMC 2018 Sep 7. (PMC6128407; doi:10.3390/pr6050042)
Supplement: Supplemental Table 2 [file NIHMS964769-supplement-Supplemental_Table_2.docx]

**Supplemental Table 2.** List of 86 miRNAs representing 72 unique seeds where ADAR editing of mRNA transcripts alters complementarity to their seed region and creates novel target sites for regulation. MiRNA information was obtained from miRBase, ‘RC’ indicates the seed is reverse complemented, ‘Edited Targets’ is the total number of seed matches when the transcripts are edited whereas ‘Unedited Targets” indicates the number of seed matches in the absence of editing activity. ‘Expected Targets’ is the average number of seed matches found within the windows flanking the edit site.

**miRNA Seed (RC) Edited Unedited Expected**

**Targets Targets Targets**

| miR-6778-3p | GGGAGGC | 161 | 21 | 4.16366 |
| --- | --- | --- | --- | --- |
| miR-3190-5p | TGGCCAG | 158 | 26 | 1.579897 |
| miR-513a-5p | CCTGTGA | 151 | 2 | 0.350515 |
| miR-450b-3p, -769-3p | GATCCCA | 149 | 2 | 0.243557 |
| miR-4691-3p | GTGGCTG | 136 | 0 | 0.45232 |
| miR-6089 | CGGCCTC | 122 | 0 | 0.704897 |
| miR-548s | TTGGCCA | 102 | 27 | 1.54768 |
| miR-3189-3p | CCCAAGG | 99 | 11 | 0.240979 |
| miR-17-3p | ACTGCAG | 95 | 25 | 0.76933 |
| miR-6813-3p | CCAAGGT | 95 | 16 | 0.398196 |
| miR-1273g-3p | GCAGTGG | 94 | 28 | 1.725515 |
| miR-3065-3p | GGTGCTG | 93 | 0 | 0.28866 |
| miR-140-3p | CTGTGGT | 91 | 0 | 0.421392 |
| miR-340-3p, -6827-3p | GAGACGG | 89 | 9 | 1.070876 |
| miR-1207-3p | CCAGCTG | 89 | 9 | 0.493557 |
| miR-3622a-3p, -3622b-3p | TCAGGTG | 83 | 38 | 0.858247 |
| miR-646 | AGCTGCT | 76 | 7 | 0.466495 |
| miR-6894-3p | GCAGGCA | 73 | 22 | 0.380155 |
| miR-4793-3p | AGTGCAG | 67 | 14 | 1.256443 |
| miR-4537 | TCGGCTC | 62 | 0 | 0.588918 |
| miR-1281 | GGAGGCG | 61 | 4 | 0.57732 |
| miR-30a-3p, -30d-3p, -30e-3p | ACTGAAA | 58 | 31 | 0.523196 |
| miR-1247-3p | TCCCGGG | 57 | 0 | 0.483247 |
| miR-129-5p | GCAAAAA | 52 | 21 | 0.439433 |
| miR-222-5p | CTACTGA | 52 | 12 | 0.274485 |
| miR-629-3p | GGGAGAA | 51 | 15 | 0.449742 |
| miR-548c-3p | GATTTTT | 50 | 8 | 0.818299 |
| miR-412-3p, -6754-3p | GGTGAAG | 49 | 6 | 0.195876 |
| miR-484, -3155a, -3155b | GAGCCTG | 44 | 8 | 0.408505 |
| miR-3680-3p | ATGCAAA | 43 | 21 | 0.403351 |
| miR-4437 | TGAGCCC | 41 | 11 | 0.615979 |
| miR-1229-3p | GGTGAGA | 39 | 5 | 0.340206 |
| miR-3192-5p | CTCCCAG | 36 | 23 | 0.93299 |
| miR-942-3p | GCCATGT | 36 | 11 | 0.518041 |
| miR-330-5p, -326 | CCCAGAG | 34 | 3 | 0.384021 |
| miR-936 | TCTACTG | 32 | 3 | 0.298969 |
| miR-514a-5p | CCAGAGT | 32 | 2 | 0.368557 |
| miR-6078 | TCAGGCG | 31 | 1 | 0.092784 |
| miR-6512-3p, -6720-5p | GGCTGGA | 29 | 5 | 1.466495 |
| miR-4314 | TCCCAGA | 28 | 5 | 0.409794 |
| miR-766-3p | GCTGGAG | 26 | 7 | 1.412371 |
| miR-6087 | CCGCCTC | 25 | 0 | 1.216495 |
| miR-34a-3p | GCTGATT | 25 | 3 | 0.268041 |
| miR-508-5p | CTGGAGT | 25 | 5 | 1.43299 |
| miR-6078 | TCAGGCG | 24 | 2 | 0.07732 |
| miR-6511a-3p, -6511b-3p | TGGTGAG | 24 | 0 | 0.262887 |
| miR-153-5p | AAAAATG | 22 | 9 | 0.930412 |
| miR-7703 | GAGTGCA | 22 | 11 | 1.087629 |
| miR-3614-5p | CCAAGTG | 21 | 4 | 0.309278 |
| miR-6771-5p | CTCCCGA | 21 | 3 | 0.93299 |
| miR-6892-3p | GAGAGGG | 20 | 7 | 0.340206 |
| miR-4634 | TCGCGCC | 20 | 0 | 0.201031 |
| miR-3667-3p | AGGAAGG | 19 | 9 | 0.5 |
| miR-374b-3p | CTGCTAA | 19 | 3 | 0.231959 |
| miR-4707-3p | GGCGGGC | 19 | 0 | 0.592784 |
| miR-197-3p | GTGGTGA | 19 | 0 | 0.340206 |
| miR-6749-3p | GGGAGGA | 19 | 2 | 0.515464 |
| miR-1248 | AAGAAGG | 18 | 7 | 0.438144 |
| miR-4539 | AGTTCAG | 18 | 8 | 0.304124 |
| miR-371a-3p | CGGCACT | 18 | 0 | 0.046392 |
| miR-149-5p | GAGCCAG | 18 | 0 | 0.546392 |
| miR-1306-5p | GGAGGTG | 18 | 2 | 0.943299 |
| miR-1199-5p, -6751-3p | GGCTCAG | 18 | 3 | 0.365979 |
| miR-331-3p | CCAGGGG | 17 | 2 | 0.242268 |
| miR-4691-5p, -6792-3p | TGGAGGA | 17 | 5 | 0.391753 |
| miR-325 | CTACTAG | 16 | 1 | 0.118557 |
| miR-1260a, -1260b | GGTGGGA | 15 | 1 | 0.721649 |
| miR-630 | AGAATAC | 15 | 1 | 0.126289 |
| miR-6752-3p | GGCAGGG | 15 | 6 | 0.407216 |
| miR-519d-5p | TTTGGAG | 13 | 4 | 0.43299 |
| miR-18a-3p | AGGGCAG | 13 | 3 | 0.309278 |
| miR-4267 | GAGCTGG | 12 | 1 | 0.319588 |
